# Unveiling Hidden Abscesses: The Clinical Utility of Diffusion-Weighted Whole-Body Imaging with Background Suppression (DWIBS) in Metastatic Abscess Screening

**DOI:** 10.3390/diagnostics16020223

**Published:** 2026-01-10

**Authors:** Koji Hayashi, Maho Hayashi, Rina Izumi, Mamiko Sato, Seigaku Hayashi, Toshiko Iwasaki, Ippei Sakamaki, Yasutaka Kobayashi

**Affiliations:** 1 Department of Rehabilitation Medicine, Fukui General Hospital, Fukui 910-8561, Japansatomoko@f-gh.jp (M.S.); 2 Department of Internal Medicine, Fukui General Hospital, Fukui 910-8561, Japan; 3 Graduate School of Health Science, Fukui Health Science University, Fukui 910-3190, Japan; yasutaka_k@fukui-hsu.ac.jp; 4 Department of Orthopedics, Fukui General Hospital, Fukui 910-8561, Japan; 5 Department of Radiology, Fukui General Hospital, Fukui 910-8561, Japan; 6 Department of Infectious Diseases, Faculty of Medical Sciences, University of Fukui, Fukui 910-1193, Japan; sakamaki@u-fukui.ac.jp

**Keywords:** abscess, metastatic lesion, prostate abscess, diffusion-weighted whole-body imaging with background suppression, MRI, methicillin-resistant *Staphylococcus aureus*, diagnosis, radiology

## Abstract

A 74-year-old man with type 2 diabetes presented with fever, urinary retention, and urinary difficulties. Initial abdominal Computed Tomography (CT) suggested acute pyelonephritis, but a low-density area in the prostate was overlooked. Following the confirmation of methicillin-resistant *Staphylococcus aureus* (MRSA) in blood and urine cultures, comprehensive screening for metastatic abscesses was necessitated. Diffusion-weighted whole-body imaging with background suppression (DWIBS) was utilized and clearly identified a prostatic abscess (PA), nephritis, urethritis, and subcutaneous cysts. These findings also raised suspicion of pyogenic vertebral osteomyelitis. Crucially, the PA, urethritis, subcutaneous cysts, and potentially the vertebral osteomyelitis were either overlooked or not detected by initial CT imaging. DWIBS allows for simultaneous whole-body screening and serves as a useful adjunctive tool for identifying minute abscesses, which may assist in detecting inflammatory foci that are sometimes overlooked by conventional imaging. Unlike CT, DWIBS avoids radiation and contrast agents, and is significantly more cost-effective than positron emission tomography-CT (PET-CT). DWIBS can thus serve as a useful, non-invasive tool for the early detection and exclusion of abscesses in other organs when metastatic abscess formation is suspected or cultures are positive for microorganisms causing metastatic abscesses.

**Figure 1:**
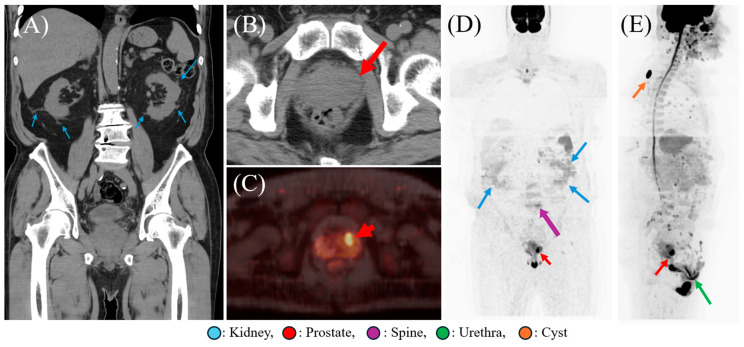
Abdominal Computed Tomography (CT) and Diffusion-Weighted Whole-Body Imaging with Background Signal Suppression (DWIBS) findings. (**A**) Abdominal CT without contrast agents demonstrates increased fat density and fuzziness around the kidneys, accompanied by perinephric fat stranding (arrows), consistent with acute pyelonephritis. A slice thickness of 4 mm was used for acquisition (Revolution^TM^ Maxima; GE Healthcare). Coronal section, soft tissue window. (**B**) Retrospective analysis of the coronal section of the abdominal CT reveals hypodensity in the prostate (arrow). Axial section, soft tissue window. (**C**) DWIBS findings clearly highlight the focus of prostate (red arrow). (**D**) DWIBS findings disclosed prostate (red arrow) and renal accumulation (blue arrows), as well as slight accumulation in the lumbar vertebrae (purple arrow). (**E**) DWIBS results revealed the high signal in the prostate (red arrow), sebaceous cyst (orange arrow) and urethral tract (green arrow). DWIBS was performed using a 1.5T MRI system (SIGNA^TM^ ARTIST EVO; GE Healthcare). DWIBS findings in (**C**) is a section of fusion images as well as (**D**,**E**) are presented as maximum intensity projection (MIP) images generated from the 3D dataset. The specific imaging protocols were described in Table S1. Original DICOM data were fully anonymized. A 74-year-old man had a history of type 2 diabetes mellitus developed fever followed by urinary retention and worsening urinary difficulties. He had undergone a routine medical checkup one month prior to symptom onset, during which his prostate-specific antigen (PSA) level was normal (3.024 ng/mL). Laboratory tests indicated an elevated white blood cell (WBC) count of 11.5 × 10^3^/μL, with neutrophils making up 84.3% of the WBC fraction. Additionally, the blood urea nitrogen (BUN) level was 23.5 mg/dL, total bilirubin was 2.4 mg/dL, and C-reactive protein (CRP) was significantly elevated at 15.57 mg/dL. Urinalysis showed strongly positive results for white blood cells, protein, occult blood, and glucose. Based on these findings, urinary tract infection (UTI) was suspected. A plane abdominal CT scan revealed increased fat density and fuzziness around the kidneys, accompanied by perinephric fat stranding, consistent with acute pyelonephritis (Figure 1A). Additionally, a retrospective analysis revealed a low-density area in the prostate; however, this finding was overlooked by both radiologists and infectious disease specialists (Figure 1B). Empirical therapy with ceftriaxone was initiated upon admission. On day 2, urinary and two sets of blood cultures tested positive for methicillin-resistant *Staphylococcus aureus* (MRSA), prompting us to screen for metastatic abscesses. We used DWIBS to comprehensively assess the presence of abscesses throughout the body. DWIBS findings clearly identified a prostatic abscess (PA), nephritis, urethritis, and subcutaneous cysts (Figure 1C–E). Additionally, the imaging raised suspicion of pyogenic vertebral osteomyelitis (Figure 1D), although this remained unconfirmed by invasive procedures. Importantly, PA, urethritis, subcutaneous cysts, and potentially the vertebral osteomyelitis were either overlooked or not detected by initial CT imaging. Echocardiogram was unremarkable. Following the identification of MRSA, therapy was switched to daptomycin on Day 2, and the patient’s clinical symptoms resolved within 10 days. Treatment was subsequently transitioned to oral trimethoprim/sulfamethoxazole on Day 14 for a 21-day course. Repeat blood cultures were obtained on Day 23, with a negative result confirmed on Day 28. All antibiotic therapy was successfully discontinued on Day 35. DWIBS was introduced by Takahara et al. in 2004, serving as a sophisticated MRI method for comprehensive whole-body cancer detection [1,2]. Utilizing a short τ inversion recovery (STIR) echo-planar imaging sequence amenable to free-breathing, it facilitates the sensitive identification of diffusion-restricted lesions, such as cancers and abscesses, throughout the entire body [2,3]. Despite its standard use, CT frequently demonstrates insufficient sensitivity for minute abscesses and carries the drawback of radiation exposure [4,5,6]. Positron emission tomography-computed tomography (PET-CT), while providing high sensitivity, is constrained by its substantial cost and associated radiation dose [4]. More recently, the diagnostic value of DWIBS has been demonstrated in various infectious and inflammatory conditions—such as myocardial abscesses, acute focal bacterial nephritis, and aortitis [3,5,6]. Differences among these modalities are summarized in Table S2. MRSA was confirmed in blood and urine cultures. MRSA is a relatively uncommon cause of pyelonephritis, which necessitated excluding abscesses in other organs. Consequently, we investigated other potential infection sources, focusing on DWIBS. Its advantage is the ability to simultaneously detect abscesses and tumors throughout the body, offering convenience similar to PET-CT [1,3,7,8,9,10,11]. Importantly, DWIBS is significantly more cost-effective than PET-CT and does not involve radiation exposure. Although DWIBS is currently primarily used for staging and tumor detection, some studies have demonstrated its application in identifying abscesses and inflammation [3,9,10,11]. Our case confirmed MRSA in blood and urine cultures, necessitating the exclusion of abscesses in other organs due to MRSA’s tendency to cause abscess formation in both primary and distant sites. However, differentiating between abscess and tumor based solely on DWIBS can be challenging. Since invasive confirmation (e.g., biopsy or drainage) was not performed for each site, the diagnoses were established based on a combination of clinical findings, laboratory data, imaging characteristics, and response to targeted therapy. Regarding the diagnostic validity for each site, PA was strongly supported by the presence of a focal cavity identified as a low-density area on CT and corresponding distinct high-signal intensity on DWIBS. The clinical diagnosis was further corroborated by practical clues, including a significant systemic inflammatory response (WBC 11.5 × 10^3^/μL; CRP 15.57 mg/dL) and the prompt resolution of high fever and urinary retention within 10 days of switching to MRSA-targeted daptomycin. Furthermore, unremarkable follow-up MRI findings (Figure 1) confirmed the complete resolution of the prostatic lesion, while a normal PSA level (3.024 ng/mL) reduced the likelihood of malignancy. Notably, the causative pathogen was MRSA, which is increasingly recognized as a major cause of PA [12]. Regarding the management of PA, surgical drainage was not performed. This clinical decision, made in consultation with urologists, was based on the patient’s prompt and favorable clinical and biochemical response to targeted systemic antibiotic therapy. Specifically, the patient’s fever and urinary symptoms significantly improved within 10 days of initiating MRSA-targeted treatment. Furthermore, follow-up imaging confirmed the resolution of the prostatic lesion without invasive intervention. Nonetheless, it is important to note that conservative management should be discontinued in favor of drainage procedures—such as aspiration or incision—if the abscess diameter exceeds 1–2 cm or if pyrexia persists through the initial 72 h of antibiotic therapy [13,14]. Acute pyelonephritis was identified through consistent findings between CT (perinephric fat stranding) and DWIBS (abnormal renal accumulation). For urethritis, DWIBS depicted abnormal signal accumulation along the urethral tract, which was likely attributable to either MRSA infection or mechanical injury from catheterization, potentially complicated by secondary inflammation. Most importantly, the involvement of the lumbar spine must be interpreted with caution. We have labeled the vertebral finding as “suspected” pyogenic vertebral osteomyelitis, as no additional confirmatory imaging or biopsy was conducted. However, the subtle signal accumulation observed on DWIBS, coupled with MRSA’s strong propensity for vertebral abscess formation, maintained a high index of clinical suspicion throughout the management period. The rapid resolution of all clinical symptoms within 10 days of MRSA-targeted therapy further supports the inflammatory nature of these multi-focal findings. Intriguingly, DWIBS also incidentally visualized a subcutaneous cyst in his shoulder. Although we consider this likely unrelated to the MRSA infection due to the complete absence of inflammatory signs (e.g., swelling, redness, pain), this incidental finding subtly underscores DWIBS’s high sensitivity and broad imaging capability, even for seemingly innocuous lesions. These observations collectively highlight the potential clinical utility of DWIBS. Its high detection capabilities for screening minute abscesses are suggestive, often revealing lesions missed by conventional imaging. Considering its cost-effectiveness, DWIBS could emerge as a useful tool—not only for the early detection of metastatic abscesses but also for guiding subsequent investigations toward the elusive infectious or inflammatory focus, thereby potentially altering therapeutic strategies and improving patient outcomes. When the source of infection remains unclear—particularly if abscess formation is suspected or cultures are positive for microorganisms causing metastatic abscesses—DWIBS may complement or extend beyond standard imaging modalities such as CT or scintigraphy. DWIBS may offer economic and non-invasive benefits, as it does not use costly radionuclides that could result in radiation exposure [8]. Unlike CT, DWIBS avoids radiation and contrast agents while enabling high-sensitivity evaluation of abscesses and distant metastatic lesions [3,9,10,11]. Moreover, as demonstrated in this case, DWIBS can assist in excluding abscesses in other organs, helping to narrow the diagnostic differential and guide targeted management. To fully realize its potential, further research is warranted to validate these preliminary findings and establish DWIBS as a fundamental tool in infectious disease diagnostics.

## Data Availability

The data presented in this study are available on request from the corresponding author. Due to patient privacy and ethical considerations, the data are not publicly accessible.

## Supplementary Materials

The following supporting information can be downloaded at: https://www.mdpi.com/article/10.3390/diagnostics16020223/s1, Table S1: The DWIBS parameter in detail. Table S2. Comparison of CT, DWIBS, and PET-CT in clinical practice.
