# Supplementary material for: Unveiling Hidden Abscesses: The Clinical Utility of Diffusion-Weighted Whole-Body Imaging with Background Suppression (DWIBS) in Metastatic Abscess Screening
Source: Diagnostics (Basel). 2026 Jan 10;16(2):223. doi: 10.3390/diagnostics16020223 (PMC12840465; doi:10.3390/diagnostics16020223)
Supplement: Supplementary file 1 [file diagnostics-16-00223-s001.zip › diagnostics-4064663-supplementary.pdf]

Table S1: The DWIBS parameter in detail

Parameter	Description
Scanner/Model	GE Healthcare SIGNA™ Artist Evo
Field Strength	1.5 Tesla
Coil	Head Neck 19 ch with Anterior Bridge, Spine Posterior 40ch, Multi Purpose (MP) Large 21 ch AIR, Body Anterior (BA) Large 30 ch AIR
b-values	1000 s/mm²
Diffusion Scheme	Echo-planar imaging (EPI) with enhanced DWI (eDWI); 3 directions; parallel imaging (ASSET)
STIR Parameters	Fat suppression via STIR (inversion time 180 ms, TR/TE 7500/90.2 ms)
Voxel Size	5 mm isotropic (4×4×5 mm³)
Breathing	Free-breathing (no respiratory triggering)
Acquisition Time	~3 min per station (whole-body: 6 stations, total ~20 min)

Table S2. Comparison of CT, DWIBS, and PET-CT in clinical practice

Modality	Coverage	Radiation / Contrast	Typical Indications	Main Pitfalls
CT	Regional or Whole-body	Ionizing radiation and often requires contrast agents	First-line screening for acute infections and inflammation as well as tumors	Insufficient sensitivity for minute abscesses [4, 6]; radiation exposure and contrast-induced nephropathy or allergy
DWIBS	Whole-body	None (No radiation or contrast needed)	Screening for tumors and cancer staging [1-3]	False positives in regions with hypercellularity or those containing specific components; sensitive to motion and susceptibility artifacts [11]
PET-CT	Whole-body	Highest radiation (X-ray + radionuclides)	Staging, tumor detection, and elusive inflammatory focus [11, 12]	Very high cost; physiological uptake in brain/liver may obscure lesions [11, 12]; limited availability
